# Association between breastfeeding support and breastfeeding rates in the UK: a comparison of late preterm and term infants

**DOI:** 10.1136/bmjopen-2015-009144

**Published:** 2015-11-13

**Authors:** Sarah Rayfield, Laura Oakley, Maria A Quigley

**Affiliations:** 1 Policy Research Unit in Maternal Health and Care, National Perinatal Epidemiology Unit, University of Oxford, Oxford, UK; 2 Nuffield Department of Population Health, University of Oxford, Oxford, UK; 3 Department of Non-communicable Disease Epidemiology, London School of Hygiene and Tropical Medicine, London, UK

**Keywords:** Public health, NUTRITION & DIETETICS, Epidemiology

## Abstract

**Objective:**

To explore the association between breastfeeding support and breastfeeding among late preterm (gestation 34–36 weeks) and term (gestation ≥37 weeks) infants.

**Methods:**

Secondary analysis of the UK 2010 Infant Feeding Survey. Logistic regression was used to determine the association of breastfeeding support with breastfeeding at 10 days and 6 weeks in late preterm and term infants.

**Results:**

The study included 14 525 term and 579 late preterm infants. A total of 11 729 infants initiated breastfeeding (11 292 (81.1%) term, 437 (79.4%) late preterm infants, p=0.425). Of these, 9230 (84.3%) term and 365 (85.6%) late preterm infants were breastfeeding at 10 days (p=0.586); of these 7547 (82.0%) term and 281 (75.4%) late preterm infants were still breastfeeding at 6 weeks (p=0.012). Mothers who reported receiving contact details for breastfeeding support groups had a higher likelihood of breastfeeding late preterm (adjusted ORs, aOR 3.14, 95% CI 1.40 to 7.04) and term infants (aOR 2.24, 95% CI 1.86 to 2.68) at 10 days and term infants at 6 weeks (aOR 1.83, 95% CI 1.51 to 2.22). Those who reported that they did not receive enough help with breastfeeding in hospital had a lower likelihood of breastfeeding late preterm at 10 days and term infants at 10 days and 6 weeks, compared to those who reported having enough help.

**Conclusions:**

Receiving sufficient help with breastfeeding in hospital and the contact details for breastfeeding support groups is associated with breastfeeding term infants up to 6 weeks and late preterm infants at 10 days.

**Strengths and limitations of this study**

- Secondary data analysis of a large national survey and one of the first studies to specifically compare the role of breastfeeding support, as perceived by the mother, in late preterm and term infants.
- Mothers were oversampled from the most deprived quintile of the Index for Multiple Deprivation in the original survey. The use of survey weights in this study allowed for the oversampling and also non-response, thereby ensuring adequate representation of this difficult to reach group.
- An extensive range of confounding factors were considered and adjusted for in the analysis including ethnicity, socioeconomic status, marital status and age mother left full time education. This is important as breastfeeding is known to be associated with a number of sociodemographic factors.
- This study is retrospective in design with the Infant Feeding Survey being completed by parental self-report of both breastfeeding support and breastfeeding duration when the infants were approximately 6 weeks old, therefore, the results of this analysis may be limited by recall bias or potentially a socially desirable response bias and it is not possible to infer causality.
- Infants classified as breastfeeding in this study are likely to represent a very heterogeneous group, ranging from primarily breastfed infants to infants receiving minimal breastmilk.

## Introduction

Infants born between 34+0 and 36+6 weeks gestation are increasingly described as ‘late preterm’.^1^ This highlights that despite their size and weight often being similar to term infants, they are physiologically relatively immature,^2^ with higher rates of morbidity and mortality^2–4^ compared to term infants. Breastfeeding protects against gastrointestinal^5–10^ and respiratory illnesses^9–12^ and is associated with better cognitive development in childhood, particularly in preterm infants.^13^ Despite potential benefits for this group, late preterm infants experience lower rates of breastfeeding initiation and continuation compared to term infants.^14^ ^15^

The WHO and UK Departments of Health recommend exclusive breastfeeding for the first 6 months.^16^ Although 81% of UK mothers initiate breastfeeding, this rapidly drops to 55% at 6 weeks and 34% at 6 months, with less than 1% of infants exclusively breastfeeding at 6 months.^17^ Predictors of breastfeeding success in high-income countries include non-white ethnicity,^18^ ^19^ increasing maternal age^20–22^ and higher age of leaving full time education.^20^ ^22^ Similar sociodemographic patterns have been shown in late preterm infants^23^ although in general, evidence regarding breastfeeding in late preterm infants is limited.

Breastfeeding support is recommended in the Baby Friendly Hospital initiative (BFHI)^24^ and can comprise professional or lay support, educational or supportive interventions, or any combination of these. Breastfeeding support is more likely to be effective if it is proactive, delivered face to face and provided on an ongoing basis.^25^ However, the majority of studies evaluating the effects of breastfeeding support are restricted to healthy term infants or they evaluate the impact of breastfeeding given in the neonatal unit (NNU) to preterm infants.^26^ In the UK, at the time of the survey, support and advice about breastfeeding is usually provided initially by midwives during pregnancy. Advice may also be offered during antenatal classes, either provided through the NHS, or by third sector organisations. After the birth of an infant, breastfeeding support would initially be provided by midwives or midwife care assistants within a hospital setting. Most hospitals offer breastfeeding support sessions including breastfeeding counsellors. At around day 10 of life, care of the mother and infant is transitioned from midwifery to the health visiting team who continue to care for the child until the age of 5 years. Health visitors can offer guidance and support with breastfeeding. In addition, breastfeeding support groups including counsellors and peer support are often provided in children's centres or by third sector organisations the details of which would be provided to the mother by either the midwife or health visitor.

Given the increased vulnerability in the late preterm population and higher likelihood of breastfeeding difficulties, it is particularly necessary to understand which breastfeeding support is the most effective for this group. The objective of this study was to investigate the association between different forms of breastfeeding support on breastfeeding rates at 10 days and 6 weeks in late preterm and term infants.

## Methods

### Study design

This was a cross-sectional study involving secondary analysis of data from the UK 2010 Infant Feeding Survey (IFS).^17^ The IFS is undertaken every 5 years in order to monitor breastfeeding prevalence and infant feeding practices. In 2010, it comprised a nationally representative unclustered sample of 30 760 infants with oversampling of mothers from the most deprived quintile of the Index for Multiple Deprivation (IMD). The use of weights in the survey analysis allowed for this oversampling and differential non-response according to mother's age and IMD, and aims to makes the survey representative of the UK population. The IFS included three postal questionnaires assessing feeding status of the infant at 6–10 weeks, 4–6 months and 8–10 months. They were completed by self-report, usually by the mother of the infant. The IFS questionnaire in 2010 was based on the previous version and was piloted in all four countries of the UK prior to being rolled out. The original survey was approved by the Ethics committee, Department of Health Sciences at the University of York.

This study primarily used data from the first questionnaire, which a total of 15 724 mothers responded to (51% response rate). This questionnaire contained 150 questions over 39 pages (see web appendix 1: Questionnaire) including questions on breastfeeding duration and breastfeeding support accessed. For infants who were less than 6 weeks at the first questionnaire, data were also used from the second questionnaire (3382 infants). The analysis was restricted to singleton births born at more than 34+0 weeks gestation (15 104 infants) (see web appendix 2: flow chart). Only those infants who initiated breastfeeding were included in the analysis of breastfeeding at 10 days. Only those still breastfeeding at 10 days were included in the subsequent analysis at 6 weeks.

### Measures

Breastfeeding support variables were identified from questions in the stage 1 questionnaire^27^ (box 1). Specific variables were chosen to reflect different times at which support was given, from antenatal and immediately following delivery, to subsequent support given at home. To fully explore the association of breastfeeding support, these variables were also chosen to reflect the different nature of support ranging from direct support such as skin to skin contact to provision of information. Outcomes included the prevalence of breastfeeding at 10 days and 6 weeks. Infants were classified as having initiated breastfeeding if any breastmilk (direct or expressed) was received after birth, even if only once.^28^ Subsequently, infants were classified as breastfed if they were receiving any breastmilk as part of their nutrition, regardless of other fluids or solids. This included infants who were regarded as ‘partial breastfeeding’, but also those who were exclusively breastfed (when no other food or drink, not even water, except breastmilk is received). Outcome timepoints were chosen as the transition from midwife care to the health visiting team occurs at 10 days postpartum and it is accepted that breastfeeding can take 6 weeks to be fully established.

Box 1 Breastfeeding support questions*

- During pregnancy, were you taught how to position your baby for breastfeeding and how to attach your baby to your breast?
- Did you receive ‘skin to skin’ contact with your baby immediately after delivery?
- How soon following delivery was your baby put to your breast?
- In the days after birth, were you given advice on how to recognise if your baby was getting enough milk?
- Were you offered support for feeding problems encountered in the hospital or birth centre?
- While you were in the hospital, birth centre or unit, did you get enough help and support with feeding your baby?
- Since leaving the hospital, birth centre or unit, were you given details of voluntary organisations or community support groups which helps new mothers with infant feeding?
- Are you aware of the National Breastfeeding helpline?
- Since leaving the hospital, birth centre or unit, were you given help or information for any feeding problems encountered at home?
- In the first 6 weeks, did you receive breastfeeding support from a voluntary organisation, peer supporter, national breastfeeding helpline or breastfeeding support group?
- In the first 6 weeks, did you receive breastfeeding support from a health professional (midwife, midwife support worker, nurse, nursery nurse, health care assistant, health visitor, children's health clinic or doctor)?
- In the first 6 weeks, did you receive breastfeeding support from a media source (books, leaflets, magazines, television, radio or the internet)?

*Answers were based on parental recall at approximately 6 weeks postpartum.

Potential confounding factors were divided into sociodemographic characteristics and pregnancy and delivery characteristics. Sociodemographic characteristics included the infant's ethnicity, maternal age, marital status, age at leaving full time education, socioeconomic status (as defined by maternal occupation using the NSSEC system^29^), and IMD^30^ as a measure of area-based deprivation. Pregnancy and delivery characteristics included parity (subdividing multiparous women by previous breastfeeding experience of more or less than 6 weeks), type of delivery, admission to the NNU, length of stay in hospital, gestation at delivery, antenatal feeding intention and peer feeding behaviour (whether they had known other mothers during pregnancy and which feeding methods they had used).

### Statistical analysis

Logistic regression was used to calculate ORs for the association between breastfeeding support and breastfeeding at 10 days and 6 weeks. Analysis of the factors associated with breastfeeding at 10 days was based on those who initiated breastfeeding. Similarly, the analysis of breastfeeding at 6 weeks was based on those who were breastfeeding at 10 days. The analysis was conducted separately for late preterm (34+0–36+6 weeks) and term (>37+0 weeks) infants and was performed in stages due to large numbers of variables: first the sociodemographic variables (model A), then the antenatal and delivery characteristics (model B), and finally the breastfeeding support variables (model C). At each stage, variables that were not statistically significant (p>0.05) were removed from the model. The remaining statistically significant variables within models A and B were combined with model C with further dropping of variables that were not statistically significant as necessary. Antenatal feeding intention and the peer feeding variables were included in the model as a priori confounders, regardless of their p value as they are recognised as being highly predictive of subsequent feeding behaviour.^21^ ^31–33^ The final multivariable model for breastfeeding at 10 days included any remaining statistically significant variables from each of models A, B and C in addition to these two variables.

These final regression models for the late preterm and term infants at 10 days were then used as the respective models for breastfeeding at 6 weeks. Outcomes are presented as adjusted ORs (aOR), with 95% CIs. STATA V.13 was used to conduct the analysis with ‘survey commands’ to take account of the weighted sample. All percentages and ORs are presented as weighted values, whereas frequencies are unweighted values.

## Results

There were 15 104 singletons born at more than 34 weeks gestation, of which 14 525 (95.9%) were full term and 579 (4.1%) were late preterm. Overall, mothers of late preterm infants had a younger age distribution, were more likely to be from a non-white ethnic group and were more deprived than mothers of full-term infants (table 1). In addition, late preterm infants were less likely to have been born by normal vaginal delivery (NVD), were more likely to be admitted to the NNU (43.4% vs 4.4% of term infants, p<0.001) and had a longer length of stay in hospital when compared to term infants (table 1).

**Table 1:** Comparison between late preterm and term population†

Variable	Late preterm infants (34–36+6)		Term infants (37+)		p Value
	N	Per cent	N	Per cent	
Total	579	4.1	14 525	95.9	
Maternal age					
<20	40	8.7	546	5.2	0.027*
20–24	97	21.5	2095	18.3	
25–29	155	26.9	4051	28.1	
30–34	158	24.2	4691	29.0	
35+	126	18.6	3092	19.5	
Ethnicity					
White	488	81.5	12 882	86.2	0.026*
Asian/Asian British	40	11.3	615	6.8	
Black/Black British	13	4.0	362	4.1	
Other	13	3.2	291	2.9	
Marital status					
Single	91	16.4	2046	15.1	0.526
Married/living together	482	83.6	12 334	84.9	
Age mother left full time education					
<16	124	24.1	2447	19.0	0.056
17–18	158	29.2	4088	29.6	
>18	291	46.7	7869	51.5	
NSSEC					
Managerial	199	30.0	5643	35.2	<0.001***
Intermediate	103	18.3	2942	19.9	
Routine + manual	159	25.2	3759	27.2	
Never worked/not classified	118	26.6	2181	17.7	
IMD quintile					
Most deprived	172	36.4	3297	27.1	<0.001***
2	113	22.4	2930	22.4	
3	101	13.6	2914	18.7	
4	100	14.9	2748	16.4	
Least deprived	92	12.5	2262	15.4	
Parity					
Primiparous	321	58.6	7050	51.3	0.002**
Multiparous who breastfed <6 weeks	142	22.1	3480	21.3	
Multiparous who breastfed >6 weeks	16	19.3	3995	27.4	
Type of delivery					
NVD	322	61.2	8808	63.5	<0.001***
Instrumental	51	7.9	2066	13.8	
Caesarean	204	31.0	3620	22.6	
Neonatal unit admission					
Yes	258	43.4	658	4.4	<0.001***
No	321	56.6	13 867	95.5	
Length of stay in hospital					
<12 h	16	3.3	1849	14.6	<0.001***
12–24 h	52	8.9	3690	26.7	
1–2 days	83	14.7	3829	24.8	
3–7 days	309	51.3	3918	24.6	
>7 days	89	15.6	143	1.0	
Not born in hospital	3	0.9	390	3.0	

*p<0.05; **p<0.005; ***p<0.001.

†Per cent rounded to 1 decimal point (weighted). Frequencies (n) are unweighted values.

IMD, Index of Multiple Deprivation; NVD, normal vaginal delivery.

Overall, 11 729 mothers initiated breastfeeding, including 11 292 (81.1%) term infants and 437 (79.4%) late preterm infants (p=0.425). The rates of breastfeeding declined rapidly in both groups: 9230 (68.4%) term infants and 365 (67.9%) late preterm infants were still being breastfed at 10 days; and 7547 (56.1%) term infants and 281 (51.2%) late preterm infant were still being breastfed at 6 weeks. Of those who initiated breastfeeding, 9230 (84.3%) term infants and 365 (85.6%) late preterm were still breastfeeding at 10 days (p=0.586). Of those breastfeeding at 10 days, late preterm infants were significantly less likely to be breastfeeding at 6 weeks than term infants (281 (75.4%) versus 7547 (82.0%), unadjusted OR 0.67, 95% CI 0.49 to 0.92, p=0.012).

Among term and late preterm infants, breastfeeding at 10 days was higher among mothers in managerial professions and in those living in the least deprived areas (table 2). In term infants there were clear patterns of increasing rates of breastfeeding with increasing maternal age and with increasing levels of maternal education. White mothers had the lowest rates of breastfeeding at 10 days and 6 weeks among all infants, whereas mothers with previous breastfeeding experience consistently had the highest rates (table 3).

**Table 2:** Descriptive analysis* of sociodemographic characteristics

Variable	Breastfeeding at 10 days (%)		Breastfeeding at 6 weeks (%)	
Gestation	34–36+6	37+	34 –36+6	37+
N (sample size)	365 (437)	9230 (11 292)	281 (365)	7547 (9230)
Maternal age (years)				
<20	85.7	64.7	61.2	63.0
20–24	76.3	76.1	68.1	70.7
25–29	82.5	82.9	69.1	80.7
30–34	92.2	88.8	84.1	85.9
35+	92.6	89.1	84.0	87.0
Ethnicity				
White	84.5	82.1	70.1	79.4
Asian/Asian British	80.8	93.2	87.1	89.8
Black/Black British	98.1	98.9	88.2	95.7
Other	100	91.0	90.8	88.4
Marital status				
Single	88.0	75.8	62.2	71.4
Married/living together	85.7	85.4	77.0	83.1
Maternal age at leaving full time education				
<16	85.1	71.9	64.8	71.8
17–18	74.2	79.0	65.4	74.5
>18	92.9	90.0	82.9	87.0
NSSEC				
Managerial	92.3	89.4	82.6	85.9
Intermediate	81.9	81.7	58.8	80.2
Routine+manual	88.4	78.2	72.7	74.8
Never worked/not classified	77.3	84.5	79.6	84.4
IMD quintile				
Most deprived	80.2	80.3	73.0	82.4
2	87.6	84.2	82.5	81.1
3	87.2	82.8	67.8	80.8
4	88.9	88.6	76.4	82.9
Least deprived	91.2	87.7	80.4	83.0

*Per cent rounded to 1 decimal point (weighted). Frequencies (n) are unweighted values.

IMD, Index of Multiple Deprivation.

**Table 3:** Descriptive analysis* of antenatal and delivery characteristics

Variable	Breastfeeding at 10 days (%)		Breastfeeding at 6 weeks (%)	
Gestation	34–36+6	37+	34–36+6	37+
N (sample size)	365 (437)	9230 (11 292)	281 (365)	7547 (9230)
Parity				
Primiparous	86.9	83.0	73.6	79.7
Multiparous who breastfed <6 weeks	62.9	65.4	63.6	69.5
Multiparous who breastfed >6 weeks	96.9	94.9	84.8	89.1
Aware of the health benefits of breastfeeding				
No	77.7	79.6	72.1	80.4
Yes	88.0	85.1	75.5	82.1
Antenatal feeding intention				
Breastfeeding	90.9	88.2	82.4	84.7
Infant formula	68.3	39.2	31.6	54.4
Combination feed	75.6	81.6	58.2	74.5
Not decided	69.8	69.8	61.0	72.8
Knew other mothers with young infants during pregnancy and their feeding methods				
Mothers who formula fed	79.6	74.9	59.6	75.5
Mothers mixed fed	85.7	85.3	80.4	81.1
Mothers who breastfed	99.2	94.3	92.0	91.2
Did not know other mothers	81.1	84.9	79.4	82.7
Type of birth				
NVD	85.0	85.0	76.1	82.4
Instrumental	97.1	83.5	71.7	82.7
Caesarean	83.4	83.1	75.4	80.3
Neonatal unit admission				
No	86.2	84.4	81.7	82.2
Yes	84.7	83.2	67.8	78.6
Length of stay in hospital				
<12 h	81.1	83.7	96.8	81.1
12–24 h	75.2	85.9	91.8	82.7
1–2 days	87.0	84.4	76.7	83.2
3–7 days	87.2	81.7	75.7	79.5
>7 days	85.5	85.8	67.9	80.2
Not born in hospital	93.9	93.9	35.1	90.9
Gestation (weeks)				
34	85.6	–	71.8	–
35	85.9	–	71.5	–
36	85.4	–	78.1	–
37	–	81.3	–	81.0
38	–	83.3	–	83.3
39	–	84.1	–	81.5
40	–	86.1	–	82.4
41	–	83.5	–	81.1
42+		84.4	–	83.1

*Per cent rounded to 1 decimal point (weighted). Frequencies (n) are unweighted values.

NVD, normal vaginal delivery.

### Breastfeeding support in term infants

In univariable analysis, all of the breastfeeding support questions in box 1 were significantly associated with breastfeeding term infants at 10 days and all except questions 10 and 11 were significantly associated with breastfeeding at 6 weeks. In multivariable analysis, mothers of term infants who reported being given advice on recognising if their infant was getting enough milk were more likely to be breastfeeding at 10 days (aOR 1.24, 95% CI 1.05 to 1.46) (table 4) compared to mothers reporting that they were not given this advice. They were also more likely to be breastfeeding at 10 days if they reported being given the contact details of community support groups (aOR 2.24, 95% CI 1.86 to 2.68), if they were aware of the national breastfeeding helpline (aOR 1.29, 95% CI 1.08 to 1.53), or if they used support from community support groups (aOR 1.30, 95% CI 1.01 to 1.68) when compared to mothers who did not report each of these breastfeeding support activities. Mothers who reported no feeding problems in hospital were more likely to be breastfeeding at 10 days (aOR 1.57, 95% CI 1.29 to 1.91) than women who reported feeding problems and had received help in hospital; among the women who experienced feeding problems in hospital, maternal report of receiving help was not associated with breastfeeding at 10 days. In contrast, breastfeeding at 10 days was less likely in mothers who reported either no feeding problems at home (aOR 0.62, 95% CI 0.50 to 0.77) or feeding problems with no support (aOR 0.58, 95% CI 0.42 to 0.80) compared with mothers who reported receiving help for feeding problems at home. Mothers who used support from healthcare professionals were less likely to be breastfeeding at 10 days (aOR 0.63, 95% CI 0.50 to 0.79 and 6 weeks (OR 0.71, 95% CI 0.57 to 0.88) compared to those who did not use support. Out of those who had encountered feeding problems, term infants were less likely to be breastfed at 6 weeks if their mother felt they had not received help at home (aOR 0.45, 95% CI 0.35 to 0.64) compared to those who felt they had received help at home for feeding problems. Mothers who reported that they were given the contact details of community support groups were more likely to be breastfeeding at 6 weeks (aOR 1.83, 95% CI 1.51 to 2.22) compared to those who reported that they were not given the contact details.

**Table 4:** Adjusted ORs† for breastfeeding support in term infants

Variable	Breastfeeding at 10 days				Breastfeeding at 6 weeks			
	N‡ (%)§	aOR^5^	95% CI	p Value	N^6^ (%)¶	aOR^5^	95% C.I	p Value
Q4††: Received advice on how to recognise if the infant is receiving enough milk								
No	5665 (81.3)	1			4367 (80.4)	1		
Yes	5536 (88.0)	1.24	1.05 to 1.46	0.013*	4809 (83.7)	1.04	0.89 to 1.22	0.608
Q5: Received help or support in hospital for feeding problems								
Help received	3052 (80.9)	1			2380 (77.1)	1		
No help received	545 (72.6)	1.07	0.75 to 1.53	0.717	357 (71.3)	0.99	0.67 to 1.45	0.943
No problems	7089 (86.4)	1.57	1.29 to 1.91	<0.001***	5973 (83.9)	1.27	1.05 to 1.53	0.015*
Not born in hospital	502 (89.4)	0.51	0.24 to 1.09	0.082	447 (88.8)	1.18	0.56 to 2.47	0.660
Q6: Received enough help and support in hospital								
Yes	7891 (86.3)	1			6636 (83.4)	1		
No	2795 (78.2)	0.61	0.50 to 0.74	<0.001***	2064 (76.0)	0.75	0.62 to 0.90	0.003**
Not born in hospital	502 (89.4)	1.00			447 (88.8)	1.00		
Q7: Received contact details of community support groups for breastfeeding								
No	2818 (71.6)	1			1812 (74.5)	1		
Yes	8408 (88.3)	2.24	1.86 to 2.68	<0.001***	7366 (83.9)	1.83	1.51 to 2.22	<0.001***
Q8: Aware of the National Breastfeeding Helpline								
No	3550 (77.3)	1			2596 (75.9)	1		
Yes	7675 (87.3)	1.29	1.08 to 1.53	0.004**	6581 (75.2)	1.01	0.85 to 1.21	0.884
Q9: Received help or information for feeding problems‡‡ encountered at home								
Yes	3433 (89.6)	1			3023 (81.2)	1		
No	673 (75.3)	0.58	0.42 to 0.80	0.001**	492 (64.6)	0.45	0.35 to 0.64	<0.001***
No problems	7175 (82.8)	0.62	0.50 to 0.77	<0.001***	5707 (84.0)	1.26	1.04 to 1.52	0.018*
Q10: Used support from community support groups								
No	9588 (83.3)	1			7721 (81.9)	1		
Yes	1702 (89.4)	1.30	1.01 to 1.68	0.043*	1509 (82.7)	1.22	0.97 to 1.53	0.086
Q11:Used support from healthcare professionals								
No	2807 (87.2)	1			2403 (86.6)	1		
Yes	8483 (83.3)	0.63	0.50 to 0.79	<0.001	6827 (80.2)	0.71	0.57 to 0.88	0.002**

*p<0.05; **p<0.005; ***p<0.001.

†Adjusted ORs with 95% CIs and p values. Adjusted for: Ethnicity, marital status, age mother left full time education, socioeconomic status, IMD, parity with previous breastfeeding experience, antenatal feeding intention, knowing other mothers while pregnant and how they fed their infants, given advice on how to recognise if infant receiving enough milk, receiving help in hospital for feeding problems, receiving enough help in hospital, given contact details of voluntary organisation or community support group, being aware of the national breastfeeding helpline, receiving support or information for feeding problems at home, using voluntary support, using support from healthcare professionals. IMD, Index of Multiple Deprivation.

‡Total Sample size of women with term infants with each individual response to each question.

§Weighted percentage of women responding to each question who were breastfeeding at 10 days.

¶Weighted percentage of women responding to each question who were breastfeeding at 6 weeks.

††Question numbers refer to breastfeeding support questions in box 1.

‡‡Feeding problems at home could include feeding problems related to either formula feeding or breastfeeding.

### Breastfeeding support in late preterm infants

In univariable analysis, only questions 6, 7, 8, 9 and 12 from box 1 were significantly associated with breastfeeding late preterm infants at 10 days and only questions 2, 3 and 12 were significantly associated with breastfeeding at 6 weeks. In multivariable analysis, as with term infants, late preterm infants were less likely to be breastfeeding at 10 days if their mother felt they did not receive enough help with feeding in hospital (aOR 0.23, 95% CI 0.09 to 0.60, p=0.003) compared to mothers who did feel they had enough help (table 5) and more likely to be breastfeeding at 10 days if their mother reported that she was given contact details for community support groups (aOR 3.14, 95% CI 1.40 to 7.04, p=0.006) compared to mothers who reported that they were not given these details. The late preterm infants who reported no feeding problems at home were significantly less likely to be breastfeeding at 10 days compared to those who had experienced problems with feeding at home and had received help for them (aOR 0.08, 95% CI 0.02 to 0.33). In contrast to term infants, no types of breastfeeding support were associated with breastfeeding late preterm infants at 6 weeks.

**Table 5:** Adjusted ORs† for breastfeeding support in late preterm infants

Variable	Breastfeeding at 10 days				Breastfeeding at 6 weeks			
	N‡ (%)§	aOR^8^	95% CI	p Value	N^12^ (%)¶	aOR^8^	95% C.I	p Value
Q6††: Received enough help and support with feeding in hospital								
Yes	321 (89.2)	1			277 (77.0)	1		
No	102 (73.6)	0.23	0.09 to 0.60	0.003**	76 (71.0)	0.57	0.24 to 1.33	0.190
Not born in hospital	12 (98.0)	2.73	0.25 to 29.9	0.411	11 (66.3)	0.27	0.07 to 1.05	0.059
Q7: Received contact details for community support groups for breastfeeding								
No	140 (73.9)	1			102 (69.2)	1		
Yes	293 (91.3)	3.14	1.40 to 7.04	0.006**	260 (77.9)	1.86	0.87 to 3.95	0.106
Q9: Received help or information for feeding problems‡‡ encountered at home								
Had help	103 (97.6)	1			100 (81.2)	1		
No help given	31 (86.6)	0.41	0.04 to 4.08	0.449	25 (76.9)	0.86	0.18 to 3.99	0.845
No problems	302 (81.8)	0.08	0.02 to 0.33	0.001**	239 (73.1)	0.69	0.29 to 1.64	0.402

*p<0.05; ** p<0.005; ***p<0.001.

†aOR with 95% CIs and p values. Adjusted for: antenatal feeding intention, knowing other mothers while pregnant and their feeding methods, type of delivery, parity with previous breastfeeding experience, receiving enough help/support in hospital, being given contact details for voluntary organisations/community support groups, receiving help/information for feeding problems at home.

‡Sample size of women with each individual response to each question.

§Weighted percentage of women responding to each question who were still breastfeeding at 10 days.

¶Weighted percentage of women responding to each question who were still breastfeeding at 6 weeks.

††Question numbers refer to Breastfeeding support questions in table 1.

‡‡Feeding problems at home could include feeding problems related to either formula feeding or breastfeeding.

aOR, adjusted ORs.

## Discussion

Our study found a statistically significantly lower prevalence of breastfeeding at 6 weeks among late preterm infants compared to term infants. When mothers reported they had received enough help in hospital and were given contact details for support groups in the community, this was associated with a higher likelihood of breastfeeding in late preterm and term infants at 10 days and also term infants at 6 weeks. Successfully breastfeeding term infants at 10 days was also associated with being given advice to recognise if the infant was receiving enough milk, awareness of the national breastfeeding helpline and using community support groups.

Strengths of this study include the use of a national, population-based survey. The analysis employed survey weights which allowed for non-response and oversampling of mothers from the lowest quintile of the IMD ensuring adequate representation of this group. Using IFS data enabled simultaneous evaluation of a wide range of breastfeeding support among late preterm and term infants. As such, this study is one of the first to compare effectiveness of support between these two groups thereby addressing a gap in current literature. However, it is of note that the original survey findings are now 5 years old. A further strength is measuring outcomes at both 10 days and 6 weeks to identify support factors impacting on clinically important postnatal time points. The most rapid decline in breastfeeding rates occurs in the first 10 days after birth,^17^ indicating that breastfeeding support could have the most to offer mothers during that time. The risk of recall bias was limited as the questionnaire was intended for when the infant was 6 weeks old. However, given the sensitive and emotive nature of breastfeeding duration, responses may have been influenced by a socially desirable response bias with mothers perhaps overestimating the length of time they achieved breastfeeding, or underplaying the support received. The IFS questionnaire is long (150 questions) potentially presenting a challenge for a parent with a newborn infant. This may have introduced a bias in those who may have been more likely to respond. In addition, this study investigated the association between the parental perception of support given, rather than the actual support given and was limited by the wording of the original survey questions. For example while we have shown that receiving enough help with breastfeeding in hospital is associated with breastfeeding term and late preterm infants at 10 days, it was not possible to further delineate what support had been received by those who felt they had received ‘enough’. However, it may be the perception that breastfeeding support is available combined with the provision of information in case of feeding problems, is important in enabling successful breastfeeding. Finally, although the overall sample size was large (n=15 104), a further potential limitation is the relatively small number of late preterm infants in the study (n=579).

Although a large number of potential confounding factors were included in the analysis, it is not possible to exclude residual confounding by unmeasured factors, for example medical problems of mother or infant. Another limitation is the likely heterogeneous nature of the breastfeeding groups, ranging from primarily breastfed infants to infants receiving minimal breastmilk. However, it was not possible to subdivide by amount of breastmilk being received. Nonetheless, given the current low rates of breastfeeding in the UK, establishing interventions which may improve any breastfeeding remains important.

Previous studies have also identified late preterm infants as having lower rates of breastfeeding than term infants^14^ ^15^ which is likely due to a combination of factors, including delayed lactogenesis^34^ and physiological immaturity resulting in a reduced sucking ability.^35^ ^36^ While late preterm infants often do not fit criteria for NNU admission, postnatal wards may not offer the additional support required to ensure successful breastfeeding. The Infant Feeding Survey report^17^ found the main reasons women stopped breastfeeding in the first weeks after birth were due to the baby not sucking, the mother having painful breasts or feeling she had insufficient milk. For women who stopped in the first 2 weeks, the factors they felt could have helped them breastfeed for longer included more support and guidance from hospital staff, midwives and families (23% of respondents), if the baby had latched on the breast easier (19%) and if there had been less pain (14%). This study found receiving enough help in hospital was associated with breastfeeding late preterm infants at 10 days and term infants at 10 days and 6 weeks. This type of support may have been delivered via a number of different mechanisms. Both Oakley *et al*^33^ and Henderson and Redshaw^32^ had previously noted associations between initial midwifery support and breastfeeding at 6 weeks and 4 months respectively, support which is likely to have taken place in the hospital. Renfrew *et al*^25^ found all forms of ‘breastfeeding support’ to have a positive impact on breastfeeding in term infants. Those results are borne out by the wide variety of support associated with breastfeeding at 10 days in the term infants in our study. The vast majority of previous studies only included healthy term infants although Henderson and Redshaw^32^ observed lower rates of breastfeeding initiation in preterm than term infants (77.2% vs 80.5%). However, they did not differentiate between the two groups when analysing the impact of breastfeeding support.

In this study, term infants whose mothers used support from health professionals were less likely to be breastfeeding at 10 days and 6 weeks. This finding is in contrast to existing evidence such as the Cochrane review,^25^ which found that breastfeeding support was associated with increasing breastfeeding duration. However, our result is likely to be confounded by the fact that those experiencing feeding problems are more likely to cease breastfeeding, but also more likely to be accessing professional support—41% of those using health professional support had also stated they had experienced feeding problems in hospital, compared to only 5% who had not used this support. Late preterm and term infants were less likely to be breastfeeding at 10 days if they had not experienced feeding problems at home. However, this may be the result of mothers who stopped breastfeeding early, potentially prior to discharge from hospital as mothers were classified as having initiated breastfeeding, even if the infant only had one breastfeed after birth. Among the late preterm infants, 37% of those with no feeding problems had already stopped breastfeeding by 5 days, compared to only 7% of those who had help for feeding problems. By 6 weeks of age, term infants were more likely to be breastfeeding if they had not encountered feeding problems at home as this analysis only included infants who were still breastfeeding by 10 days.

This study identified that late preterm and term infants were more likely to be breastfeeding at 10 days if their mother had received the contact details for a community support group, compared to women who did not receive these details. However, it was not possible to determine whether these women actually attended a support group or not and at what age of the infant. A cluster randomised trial^37^ of a policy to provide breastfeeding groups in primary care found increased provision of breastfeeding groups did not improve breastfeeding rates at 6–8 weeks for the intervention group compared to the control group. The median infant age for attending such a group in this trial was 5 weeks, which would be counter to the fact that receiving this information was associated with breastfeeding at 10 days in our study. It is of note, however, that the trial was introducing breastfeeding support groups into relatively deprived areas of Scotland which may have impacted the results as it is well recognised that increasing deprivation is associated with lower rates of breastfeeding.

Previous studies have demonstrated strong associations between sociodemographic factors and breastfeeding^21^ ^22^ ^31–33^ ^38^ which our study replicated among term infants. Demirci *et al*,^23^ with a large sample of 7012 late preterm infants found breastfeeding initiation was associated with maternal education, marital status and ethnicity. In our study no sociodemographic characteristics were statistically significant in late preterm infants which was in contrast to the findings for term infants. Although our sample size was relatively small, and may not have had sufficient power to identify particular effects, this finding may also suggest that sociodemographic factors may be less relevant in determining continuation of breastfeeding in late preterm infants compared to term infants.

Breastfeeding rates in the UK have a long way to go before reaching the WHO recommendations and it is clear that providing breastfeeding support will be part of this. This study has identified that while a similar proportion of term and late preterm infants initiated breastfeeding, the late preterm infants were significantly less likely to be breastfeeding by 6 weeks. Relatively simple breastfeeding support methods, such as the provision of contact details for community breastfeeding support groups has been demonstrated by this study as being associated with successful breastfeeding for term and late preterm infants. Mothers who felt they had received enough help and support with breastfeeding while in hospital were also more likely to be breastfeeding late preterm and term infants at 10 days. Support for breastfeeding should be at the forefront of maternity practice in hospital, and community services need to ensure that basic information on how to obtain help and support is visibly given to all mothers while instituting a culture of easily accessible breastfeeding support for mothers if and when they need it.

In conclusion, our study found breastfeeding support to be positively associated with breastfeeding at 10 days in late preterm and term infants and at 6 weeks in term infants, in particular receiving the contact details for community support groups and receiving enough help with breastfeeding in hospital. Increasing rates of breastfeeding should be a public health priority globally, but especially in the UK where exclusive breastfeeding rates are particularly low. Late preterm infants are likely to require additional support, given their even lower rates of breastfeeding. Further research is required on breastfeeding continuation in late preterm infants in order to understand the complex interplay of factors determining breastfeeding success for this population.
